# Correlates of objectively assessed physical activity and sedentary time in children: a cross-sectional study (The European Youth Heart Study)

**DOI:** 10.1186/1471-2458-9-322

**Published:** 2009-09-07

**Authors:** Andreas Nilsson, Lars Bo Andersen, Yngvar Ommundsen, Karsten Froberg, Luis B Sardinha, Karin Piehl-Aulin, Ulf Ekelund

**Affiliations:** 1 School of Health and Medical Sciences, Örebro University, Örebro, Sweden; 2 Department of Sports Medicine, Norwegian School of Sport Sciences, Oslo, Norway; 3 Department of Coaching and Psychology, Norwegian School of Sport Sciences, Oslo, Norway; 4 Institute of Sports Science & Clinical Biomechanics, University of Southern Denmark, Odense, Denmark; 5 Faculty of Human Movement, Technical University of Lisbon, Lisbon, Portugal; 6 MRC Epidemiology Unit, Cambridge, UK

## Abstract

**Background:**

Identifying leisure time activities performed before and after school that influence time in physical activity (PA) and/or time spent sedentary can provide useful information when designing interventions aimed to promote an active lifestyle in young people. The purpose of this study was to examine associations between mode of transportation to school, outdoor play after school, participation in exercise in clubs, and TV viewing with objectively assessed PA and sedentary behaviour in children.

**Methods:**

A total of 1327 nine- and 15-year-old children from three European countries (Norway, Estonia, Portugal) participated as part of the European Youth Heart Study. PA was measured during two weekdays and two weekend days using the MTI accelerometer, and average percent of time in moderate-to-vigorous PA (MVPA) and time spent sedentary were derived. Potential correlates were assessed by self-report. Independent associations between self-reported correlates with percent time in MVPA and percent time sedentary were analysed by general linear models, adjusted by age, gender, country, measurement period, monitored days and parental socio-economic status.

**Results:**

In 9-year-olds, playing outdoors after school was associated with higher percent time in MVPA (P < 0.01), while participation in sport clubs was associated with higher percent time in MVPA (P < 0.01) in 15-year-olds. No associations with percent time sedentary were observed in either age group.

**Conclusion:**

Frequency of outdoor play after school is a significant correlate for daily time in MVPA in 9-year-olds, while this correlate is attenuated in favour of participation in sport and exercise in clubs in 15-year-olds. Targeting walking to school or reduced TV viewing time in order to increase time in daily MVPA in children is unlikely to be sufficient. Correlates related to time spent sedentary need further examination.

## Background

Given the recent increased prevalence of overweight and obesity in youth [1-3], actions to promote physical activity (PA) and reduce time being sedentary in young people have become a public health issue. To achieve this, knowledge of influences on PA behaviour is required. PA is influenced by a broad range of correlates at different levels (e.g. socio-demographic, environmental, individual behavioural correlates) [4,5]. While some correlates (e.g. age and ethnicity) can aid when identifying groups for PA interventions, examining the potential impact of modifiable correlates on daily PA levels in children is important for developing successful interventions. For example, evaluating the relative contribution of behavioural correlates (e.g. mode of transportation to school) on daily time in PA and time spent sedentary can aid when establishing appropriate PA policies and programs for school-aged youth.

Previous studies [6,7] have showed that the majority of time during school recess and PE lessons is spent at lower intensities of PA, and the amount of time spent in moderate-to-vigorous PA (MVPA) is insufficient in relation to the recommended amount of 60 daily minutes in MVPA [8]. These findings emphasise the role of leisure time activities contributing to overall daily PA levels in children. Several leisure time behaviours hold potential to influence PA and sedentary behaviour. Unstructured outdoor play [9-11], participation in organized sports [7,12] and mode of transportation to school [13-15] have all been suggested correlates of PA levels in children and are thus possible targets for PA interventions. Conversely, time watching TV during leisure time represents a behaviour often used as a measure of sedentary time. TV viewing has been shown to be positively related to overweight in youth [1,16,17] and reducing sedentary behaviour by limiting screen time to no more than two hours per day has been recommended [18].

The influence of unstructured outdoor play, organized sports, mode of transportation to school and TV viewing on objectively measured daily time of MVPA and time spent sedentary in different age groups remain inconclusive. Previous studies evaluating influences on daily PA differ in sample size, age groups, and assessment method of PA (i.e. objective or subjective). Self-report methods can provide detailed information of the major behaviours children engage in. However, free play in young children is characterized by intermittent episodes of PA of short durations and varying intensities [19], which may not be captured by self-report. Therefore, studies including objective measurements of physical activity and sedentary behavior is likely to be better placed when examining the correlates of physical activity.

The aim of this study was to investigate the associations between mode of transportation to school, outdoor play after school, participation in sport and exercise in clubs, and TV viewing with objectively assessed time in MVPA and sedentary behaviour on a large sample of 9- and 15-year-old children.

## Methods

### Subjects

This study includes data on 9- and 15-year-old children who participated in the European Youth Heart Study (EYHS). The aims of EYHS include studies of the nature, strength and interactions between personal, environmental and lifestyle influences on cardiovascular disease risk factors in children from culturally different areas in Europe. Detailed descriptions of design, sampling procedures and assessed data variables of EYHS have previously been described [20]. In short, the study population was randomly selected using a 2-stage cluster sample procedure, with schools as primary sampling units, in geographically defined areas in four European countries (Denmark, Portugal, Estonia and Norway). A standardized protocol for data assessment was followed in all participating countries. All Danish children were excluded from the current data analysis since self-reported data on frequency in outdoor play after school and participation in sports and exercise in clubs were unavailable. A total of 2019 children participated. Only children with complete data on objectively measured PA and self-reported measures of correlates of PA were included in the analyses, which yielded a total of 762 9-year old children (Portugal: 231 [114 boys]; Estonia: 253 [126 boys]; Norway 278 [144 boys], and 565 15-year old children (Portugal: 162 [79 boys]; Estonia: 271 [100 boys]; Norway: 132 [54 boys]). There were no differences in height (9 yrs: p = 0.17; 15 yrs: p = 0.68) or weight (9 yrs: p = 0.66; 15 yrs: p = 0.95) between these children and those excluded. Written informed consent was obtained from the parent or legal guardian of each participant. The study protocol was performed in accordance with the Helsinki Declaration on biomedical research, and was approved by local research ethics committees within the study regions (Regional Committee for Medical Research Ethics, Oslo, Norway; Ethics Review Committee on Human Research of the University of Tartu, Tartu, Estonia; Ethics Committee of University of Madeira, Madeira Island, Portugal.).

### Anthropometry

Height was measured to the nearest 0.5 cm with a portable Harpenden stadiometer, and weight was measured to the nearest 0.1 kg with a beam balance scale.

### Assessment of physical activity

PA was measured using the MTI accelerometer, model 7164 (Manufacturing Technology Inc., Fort Walton Beach, FL). The children wore the accelerometer using an elastic belt attached on the hip during all waking hours for four consecutive days, including two weekdays and two weekend days. Four days was selected to combine logistic feasibility when administering to a large population and yet provide an acceptable measure of habitual PA level during an average week. Three to four days of measurement have been shown to produce a reliability coefficient between 0.7 to 0.8 compared to measuring all days of the week [21,22]. All children were verbally instructed on how to use the accelerometer the day before the start of the measurement. The accelerometer was set to record PA data every minute (60 s epoch). After downloading to a computer, a special written programme (MAHUFFE http://www.mrc-epid.cam.ac.uk/Research/PA/Downloads.html) was used to analyse the raw outcome data. Sequences of 10 or more consecutive zero counts were defined as missing data. Inclusion criteria were a minimum of three days of registration, including at least one weekend day, and at least 600 registered minutes per day. Of the 1327 children included in the present study 22% provided three complete days (i.e. ≥ 600 min each day) and 78% provided four days. Time (min·d^-1^) spent sedentary was calculated as time (min) below 100 counts per minute. Measurements during sitting and playing video games has previously been shown to produce average values below 100 counts per minute in children and adolescents [23], and this count threshold for time spent sedentary has been used in previous studies [24]. Time in moderate-to-vigorous PA (MVPA) was defined using a threshold of > 2000 counts·min^-1^. This threshold roughly estimates a walking pace of 4 km/h in youth [23,25,26], and has been used in previous studies for defining time in MVPA [27,28]. Data on time spent sedentary and in MVPA were averaged per day and expressed in relation to average monitor wear time per day. Data collection followed the school year, with the majority of measurements performed between September and May.

### Self-reported measures

Within the frame of the EYHS data collection, all participants completed a structured computerized questionnaire on numerous items related to health behaviour. The questionnaire was designed to capture behavioural, social and environmental influences on PA in children, and was partly based on a previously validated self-report instrument [29]. All children completed the questionnaire individually and a researcher was available at all times to assist if the child wanted help to correctly understand any question. Otherwise the researcher was instructed not to interfere during the completion of the questionnaire. Questions were asked individually and each question had to be answered before proceeding to the next question. For this study, the following four items was included to act as potential correlates of objectively measured percent of time being sedentary and percent time in MVPA: a) usual mode of travel to and from school b) frequency in taking part in outdoor play after school, c) participation in exercise within sport clubs, and d) daily time spent watching television.

Mode of travel to school was a closed question with three possible answers (motorized transport, bicycling and walking). However only motorized transport (non-active) and walking (active) were included in the analysis since vertical accelerometry most likely will underestimate PA from bicycling. Notably, only three 9-year-olds and eleven 15-year olds reported bicycling to school. Those who reported motorized transport in one direction and walking in the opposite direction were classified as active commuters. Data on frequency of outdoor play during leisure time ('how often do you play games outside after school?') and participation in exercise in clubs ('how often do you take part in exercise in clubs?') had four possible answers ('never/hardly ever', 'once or twice a week', 'most days', 'every day'). Before analysis, data were combined into three outcomes ('never/hardly ever', 'once or twice a week' or 'most days/every day'). Data on time watching television was based on two questions ('how many hours of TV do you usually watch before school?' and 'how many hours of TV do you usually watch after school?') with five possible answers ('none', 'less than 1 hour', '1-2 hours', '2-3 hours', 'more than 3 hours') for each question. Data on TV viewing before and after school were summarized and combined into three outcomes ('less than 2 hours per day' or '2-3 hours per day' or 'more than 3 hours per day'). In addition, data on parental socio-economic status (SES) was collected through a questionnaire to parents. The SES variable was assessed by summing the score for parents' educational level (four categories) and the mean score for parents' income (eight categories). A detailed description of the parental questionnaire regarding categories for income and education has been published elsewhere [30].

### Statistics

Mean ± SD are used to describe anthropometric measures and mean percent time in MVPA and percent time spent sedentary. Differences in anthropometric measures, mean percent time in MVPA and percent time spent sedentary between age and gender groups were tested by one-way analysis of variance (ANOVA). Differences in proportions between age and gender groups for self-reported variables were tested by chi-square (χ^2^) analysis. Univariate general linear models were used to examine the association of different correlates (mode of transportation, outdoor play after school, participation in sport clubs, and TV viewing) on mean percent time in MVPA and percent time sedentary, respectively. Time period of data collection was summarized into three defined seasons: Autumn (Sep-Nov), winter (Dec-Feb) and spring (Mar-Jun) included as a covariate, together with number of monitored days (three days or four days). All analyses were stratified by age and included gender, study location, season, monitored days and parental SES as covariates. To investigate whether gender modified the associations the interaction term (gender × correlate) was included in each model. We adjusted for study location but chose not to further investigate potential interaction terms for this variable, as it would require the analysis to go beyond the scope of the present study aim. Impact of each correlate on the dependent variables was analyzed separately. Estimates of effect sizes from significant correlates were expressed as partial Eta squared (η_p_^2^), which describes the proportion of total variability in the dependent variable attributable to the factor. Transformations for normality were performed on percent time in MVPA due to positive skewness. All data were analysed by SPSS (Statistical Package for the Social Sciences for Windows, 14.0, 2005, SPSS Inc., Chicago. IL) and alpha was set at 0.05.

## Results

Mean age was 9.6 ± 0.4 years and 15.5 ± 0.5 years in the two age groups. Mean height and weight by age and sex is shown in Table 1.

**Table 1:** Mean (± SD) height and weight stratified by age and sex.

	9-year-olds			15-year-olds		
	
	Boys	Girls	Total	Boys	Girls	Total
	(n = 384)	(n = 378)	(n = 762)	(n = 233)	(n = 332)	(n = 565)
Height (cm)	138 ± 7	138 ± 7	138 ± 7	173 ± 8*	164 ± 6	168 ± 8
Weight (kg)	33 ± 6	33 ± 7	33 ± 7	62 ± 11*	56 ± 8	58 ± 10

*Significant difference between boys and girls (P < 0.01)

The proportion of children engaging in self-reported physical activity and sedentary behaviour by age and sex is shown in Table 2. Significant differences between age groups were observed for all variables. Active commuting, outdoor play and exercise in clubs were more frequently reported in 9-year-olds compared to the 15-year-old group. A larger proportion of 9-year-olds also reported less than two hours of television viewing per day compared to the older age group. Significant gender differences were observed regarding outdoor play after school, which was more prevalent in boys than girls in both age groups. Finally, age-specific gender differences were also observed, where a larger proportion of 9-year-old girls reported less than two hours of daily TV viewing compared to boys, and a higher prevalence of 15-year-old boys reporting participation in organized sports compared to girls (Table 2).

**Table 2:** Proportion (%) of children engaging in self-reported physical activity and sedentary behaviour by age and sex

	9-yr-olds			15-yr-olds			
	Boys (n = 384)	Girls (n = 378)	Total (n = 762)	Boys (n = 233)	Girls (n = 332)	Total (n = 565)	P-value
**Transportation mode**							
Motorized	25%	27%	26%	46%	42%	44%	P < 0.01**
Walking	75%	73%	74%	54%	58%	56%	
**Exercise in clubs**							
Never/hardly ever	46%	47%	46%	46%	52%	50%	P < 0.05**
Once or twice per week	30%	33%	31%	22%	26%	24%	P < 0.05^#^
Most days of the week	24%	20%	23%	32%	22%	26%	
**Outdoor play after school**							
Never/hardly ever	18%	33%	26%	20%	36%	29%	P < 0.01**
Once or twice per week	31%	31%	31%	42%	41%	41%	P < 0.01*^#^
Most days of the week	51%	36%	43%	38%	23%	30%	
**Television viewing**							
< 2 hour per day	47%	57%	52%	46%	43%	44%	P < 0.01**
2-3 hours per day	37%	27%	32%	32%	37%	35%	P < 0.05*
>3 hours per day	16%	16%	16%	22%	20%	21%	

**Difference in proportions between age groups

*Difference in proportions between genders in 9-year-olds

^#^Difference in proportions between genders in 15-year-olds

Results on mean percent time in MVPA and time sedentary are showed in Table 3. Nine-year-olds were significantly more active at the MVPA intensity level and spent significantly less time being sedentary compared with the older group. Boys were significantly more active and spent significantly less time being sedentary compared with girls in both age groups (Table 3).

**Table 3:** Mean percent time in MVPA and time spent sedentary stratified by age and sex.

	9-year-olds			15-year-olds			
	
	Boys	Girls	Total	Boys	Girls	Total	P-value
	(n = 384)	(n = 378)	(n = 762)	(n = 233)	(n = 332)	(n = 565)	
MVPA (% time)	14 ± 7	10 ± 5	12 ± 6	12 ± 5	8 ± 4	10 ± 5	P < 0.01*^#^
Sedentary (% time)	41 ± 11	43 ± 10	42 ± 11	55 ± 13	60 ± 12	58 ± 12	P < 0.01*^#^

*Significant difference between age groups

#Significant difference between genders within both age groups

### Analysis of associations

In 9-year-olds, outdoor play after school was significantly related to a higher mean percent time in MVPA (P < 0.01; effect size [η_p_^2^] = 0.016). A gender interaction for this association was observed (P < 0.05). After stratification by gender the significant association between outdoor play and percent time in MVPA remained in both boys (P < 0.01; effect size [η_p_^2^] = 0.030) and girls (P < 0.05; effect size [η_p_^2^] = 0.023). No other self-reported correlate was significantly associated with percent time in MVPA in this age group.

In 15-year-olds, participation in sport and exercise in clubs was significantly related to a higher percent time in MVPA (P < 0.01; effect size [η_p_^2^] = 0.033). No interaction by gender was observed. No other self-reported correlate was significantly associated with percent time in MVPA in 15-year-olds.

In both age groups, no associations were observed for any of the correlates in relation to percent time sedentary.

## Discussion

The present study examined associations between selected behavioural correlates of PA with objectively measured time in MVPA and time spent sedentary in European children of different ages. Our results suggest that associations between outdoor play after school hours, sports participation and percent time in MVPA vary depending on age group of the child. The different influence of the correlates on time in MVPA between age groups was also reflected by age group differences in self-reported participation in outdoor play and organized sports. Our results also indicate that correlates significantly associated with time in MVPA (i.e. organized sports or outdoor play) do not influence on time spent sedentary, suggesting that correlates associated with sedentary behaviour is likely to differ from those associated with physical activity.

The different influence of the correlates on time in MVPA between age groups may at least partly be explained by differences in types of activities undertaken during leisure time. A significantly larger proportion in the younger age group reported outside play on most days or every day compared to the older group (Table 2). Based on observation, young children's play has been showed to incorporate intermittent episodes of PA including short bursts of vigorous intensity PA [19]; an activity pattern also observed during whole-day measurements using accelerometry [31]. A possible shift from unstructured play activities towards less intensive pursuits during leisure time is likely to make sport club participation a more important contributor to daily time in MVPA with increasing age. The observation that daily MVPA decreases with age [32], also indicated in this study (Table 3), would be in accordance with such a change in PA behaviour.

Even though no significant influence of participation in sport clubs on time in MVPA was observed in the younger age group in the present study, participation in organized sports could be justified as it may help to establish healthy lifestyle habits that persist into older age [33]. Given that participation in unstructured outdoor play decreases by age, school physical education probably becomes important for promoting a physically active lifestyle among older children who do not participate in organized sports during leisure time.

While other studies based on accelerometer measurements have estimated the contribution from active commuting to be between 5 to 24 minutes of daily MVPA [13,14], no associations between active commuting and percent time in MVPA was observed in this study. Different thresholds used when defining time in MVPA from accelerometry may contribute to the observed differences between the present and previous studies [13,14]. Nevertheless, if active commuting truly is an important contributor to the total time in MVPA, a significant association would be expected in the present analysis. Furthermore, the fact that the greater amount of time in MVPA among the active commuting children observed in previous studies [13,14] is not necessarily explained by commuting alone make conclusions about the actual influence of active commuting on daily MVPA time uncertain. We classified children who reported walking in one direction and motorized transport in the other direction as 'walkers'. Since inclusion of these children in the 'walking group' hypothetically may dilute an influence from active commuting, we reanalysed our data excluding these children, and the results were unchanged (data not shown). Thus, using active commuting as a single strategy to substantially increase average daily time in MVPA in children is unlikely to be sufficient. Notwithstanding this, as a change from motorized transport to active commuting likely will increase overall physical activity level (i.e. total energy expenditure), promoting active commuting throughout childhood and into adulthood should still be a target for public health.

The lack of an association between time in MVPA and TV viewing supports previous findings in the literature, where one review and one meta-analysis concluded that the associations between TV viewing and PA behaviour in youth are either small or close to zero, depending on the age groups studied [4,17]. The lack of association between TV viewing and sedentary time supports previous suggestions that TV viewing may not be the major determinant for being sedentary in youth [34]. Indeed, no significant associations between any of our correlates with time spent sedentary were observed. Sedentary time represented a large part of the total monitored time in both age groups (Table 3), which is in accordance with previous observations [24]. Apparently, children may accumulate substantial amounts of sedentary time, in combination with participation in activities of moderate and vigorous intensity. Studies using more comprehensive data collection procedures including identification of types of activities when examining determinants for sedentary behaviour in youth are needed.

Study strengths include the objective assessment of both time in MVPA and time spent sedentary, and the large sample of children representing various geographical and economic locations in Europe. The accelerometer has previously been proven to be a reliable tool for assessing level of PA [35] and to reflect different intensities of PA in children [31]. However, the following limitations should be acknowledged when interpreting the results. Because of the cross-sectional design, causality cannot be determined. Further, it is possible that additional factors other than age, gender, study location, season, number of monitored days and parental SES may affect observed relations between correlates and dependent variables. As all correlates were assessed by self-report, misclassification of correlate exposures may be present. The effect sizes observed for significant correlates in this study were small, which may be due to a dilution of the true effect related to non-differential misclassifications.

## Conclusion

Frequency of outdoor play after school is a significant correlate for daily time in MVPA in 9-year-olds, while in 15-year-olds participation in sport and exercise in clubs is significantly associated with time spent in MVPA. These observations indicate a transition in PA behaviour, likely due to a shift of leisure time activities towards less intensive pursuits with increasing age. Mode of transportation to school and TV viewing time were not significantly associated with time in MVPA. Thus, targeting either of these behaviours alone in order to increase time in daily MVPA in children is unlikely to be sufficient. Finally, behavioural correlates associated with time in MVPA seem not to influence time spent sedentary, suggesting that correlates associated with sedentary behaviour is likely to differ from those associated with physical activity.

## Competing interests

The authors declare that they have no competing interests.

## Authors' contributions

AN participated in design, data analysis and writing of the manuscript; LA in data analysis, statistical expertise and manuscript revision, YO in data analysis and manuscript revision, KF in design, data acquisition and manuscript revision, LS in design, data acquisition and manuscript revision, KP-A in data analysis and manuscript revision, and UE in design, data analysis and manuscript revision. All authors read and approved the final manuscript.

## Pre-publication history

The pre-publication history for this paper can be accessed here:

http://www.biomedcentral.com/1471-2458/9/322/prepub
